# Treatment of *Listeria monocytogenes* bacteremia with oral levofloxacin in an immunocompromised patient

**DOI:** 10.1016/j.idcr.2023.e01680

**Published:** 2023-01-07

**Authors:** Yo Ishihara, Kenichiro Akazawa

**Affiliations:** Department of General Internal Medicine, Shonan Kamakura General Hospital, Kamakura, Japan

**Keywords:** Bacteremia, Levofloxacin, Listeria, Listeriosis, Immunocompromised host

## Abstract

A 72-year-old woman presented with fever, malaise, and diarrhea. The patient was conscious, and was negative for meningeal signs on physical exam. Blood tests revealed elevated C-reactive protein (CRP) and white blood cell count with neutrophil dominance. Suspecting a bacterial infection, empirical antimicrobial treatment with oral levofloxacin was initiated after collecting two sets of blood culture. On the 3rd day, the patient's fever resolved. On the 7th day, *Listeria monocytogenes* bacteremia was diagnosed with both blood cultures turning positive. On the 15th day, the patient’s symptoms had improved. We ceased treatment when the CRP level decreased. *Listeria monocytogenes* is a gram-positive bacterium that causes serious infections in elderly and immunocompromised hosts. Penicillin, ampicillin, amoxicillin, and gentamicin are recommended for the treatment of *Listeria* infections. It has been reported that new fluoroquinolones may be effective *in vitro* and in animal models. Although further evidence is required, new fluoroquinolones, especially levofloxacin, may provide an option for the treatment of *Listeria* infection.

## Introduction

*Listeria monocytogenes* is a bacterium that invades the cytosol of cells and causes gastroenteritis, often resulting in debilitating effects in healthy adults. It can cause life-threatening invasive infections especially in pregnant women, newborns, the elderly, transplant recipients, and immunocompromised patients. *L. monocytogenes* are naturally present in moist environments, soil, water, and decaying vegetation and animals. Moreover, they survive and grow when subjected to refrigeration and other food preservation measures. It is often transmitted to humans through unpasteurized milk, sausages, and raw meat [1]. The most common type of *Listeria* infection is the infection of the central nervous system (CNS), followed by bacteremia or sepsis, with a mortality rate of up to 70 % [2]. *Listeria* sepsis or bacteremia accounts for one-third of adult cases of invasive listeriosis, with symptoms typically manifesting as fever and chills [3]. Beta-lactam antibiotics, such as penicillin, ampicillin, amoxicillin, and gentamicin have bactericidal effects and are recommended for the treatment of listeriosis [4]. Recently, fluoroquinolones have also been reported to be effective against *L. monocytogenes in vitro* and in animal models [5], [6], [7]; however, there are few clinical case reports. Here, we describe a case of successful treatment of *Listeria* bacteremia with oral levofloxacin in an immunosuppressed patient.

## Case

A 72-year-old woman arrived at the emergency department complaining of a fever (39 °C), malaise (since 4 days), and watery diarrhea (since 5 days). She had eaten sausages prior to the fever; however, detailed information regarding the date or type of sausage was unclear. She had a history of rheumatoid arthritis, controlled with prednisolone (5 mg/day) and methotrexate (16 mg/week).

## Clinical course

**Fig. 1:**
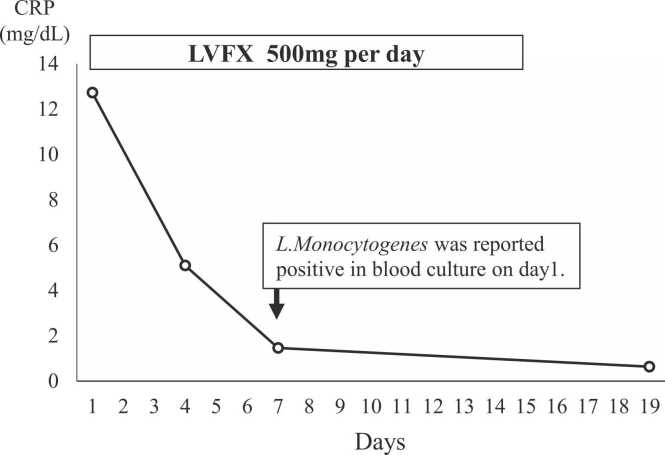
C-reactive protein (CRP) during antibiotic therapy. The patient presented with high CRP level on day 1; CRP levels decreased after 15 days of oral antibiotic therapy. CRP, C-reactive protein.

On initial evaluation, the patient was conscious, had a fever of 38.3 °C, and had tachycardia with a heart rate of 120 beats per minute. Physical examination revealed that nuchal rigidity, jolt accentuation, and Kernig sign were negative. The patient informed not being in contact with any sick individuals but recollected having eaten sausages a few days before the fever. Blood tests revealed an elevated C-reactive protein (CRP) level of 12.72 mg/dL and increased white blood cell count of 8,600/µL with neutrophil dominance (Table 1). Liver enzymes, kidney function, and coagulation factors were within normal limits. The urinary culture was negative for bacteria. Because the patient did not show meningeal signs, we did not perform a lumbar puncture. Non-enhanced computed tomography of the chest and abdomen showed no abnormalities. Considering that the patient was immunocompromised, we collected two sets of blood culture. Suspecting bacterial infection, empirical antimicrobial treatment with oral levofloxacin (500 mg/day) was initiated. The patient's fever resolved on the 3rd day of treatment. *L. monocytogenes* bacteremia was diagnosed on the 7th day with a positive blood culture, which was susceptible to levofloxacin, ampicillin, erythromycin, minocycline, and vancomycin. On the 15th day, as the CRP level had decreased to 1.46 mg/dL, we ceased treatment (Fig. 1). Because the patient’s symptoms had improved, blood cultures were not collected again. In addition, as the patient appeared to be healthy after the end of treatment, the follow-up was terminated.

**Table 1:** Laboratory data on day 1.

Hematology			Chemistry		
White blood cells	8,600	/µL	Total bilirubin	0.4	mg/dL
Lymphocyte	7.9	%	Total Protein	5.6	g/dL
Neutrocyte	84.9	%	Albumin	2.7	g/dL
Eosinocyte	0.0	%	Aspartate aminotransferase	18	U/L
Basocyte	0.1	%	Alanine aminotransferase	16	U/L
Monocyte	7.1	%	Lactate dehydrogenase	234	U/L
Red blood cells	365	× 10^4^/µL	γ-Glutamyl transpeptidase	21	IU/L
Hemoglobin	11.8	g/dL	Alkaline phosphatase	176	IU/L
Hematocrit	35.0	%	Blood urea nitrogen	17.6	mg/dL
Mean corpuscular volume	95.9	fL	Serum creatinine	0.69	mg/dL
Platelets	23.2	× 10^4^/µL	Estimated glomerular filtration rate	63.1	mL/min/1.73 m^2^
			Hemoglobin A1c	5.6	%
			C-reactive protein	12.72	mg/dL
			Procalcitonin	0.04	ng/mL

## Discussion

Here, we describe a case of *L. monocytogenes* bacteremia in an immunocompromised patient who was successfully treated with oral levofloxacin. In this case, levofloxacin, which had been prescribed as an empiric therapy for suspected bacterial infections, was suitable for treatment.

*Listeria* bacteremia causes flu-like symptoms, such as febrile illness and diarrhea, without local symptoms. Cases of bacteremia complications in CNS listeriosis are relatively high, at about 40 %, and are high-risk cases with a high mortality rate. Patients with *Listeria* infection in the CNS exhibit altered mental status, fever, seizure, focal neurological symptoms, and nuchal rigidity approximately 60 % of the time [1], [8]. In this case, the patient showed no signs of altered mental status and rigidity; thus, CNS listeriosis was thought to be negative. It was assumed that ingestion of contaminated sausages caused infection via the intestinal tract; however, stool cultures were not performed as her diarrhea had resolved.

*L. monocytogenes* is a facultative cellular, intracellular parasitic bacterium; therefore, the ideal antimicrobial agent should penetrate host cells and bind to intracellular targets. Penicillin, ampicillin, and amoxicillin are frequently used for treating *Listeria* infections. Combination therapies, such as adding gentamicin to ampicillin, have been reported to be most effective against this bacterium *in vitro*; however, gentamicin is not active against intracellular bacteria [1]. Rifampin, vancomycin, linezolid, and carbapenems are used as alternatives, and trimethoprim is used in case of intolerance of beta-lactams [9]. Gentamicin in combination with ampicillin remains the primary treatment; however, over 30 % of patients fail antibiotic therapy, therefore further secondary treatments are being considered [10]. New fluoroquinolones, such as levofloxacin, clinafloxacin, sparfloxacin, and moxifloxacin, have been reported to penetrate intracellularly, be concentrated in the cytoplasm of host cells, and show bactericidal effect against *L. monocytogenes* in multiple studies *in vitro* [11], [12], [13]. Among the new fluoroquinolones, levofloxacin was the most active against *L. monocytogenes in vitro* [5]. In animal models, the efficacy of levofloxacin against *L. monocytogenes* has been demonstrated in mice [6]. A clinical case report of levofloxacin being effective against monocytogenes meningitis also exists [14]. However, no large-scale clinical studies have been conducted. Additional clinical studies are necessary to provide further evidence of the effectiveness of levofloxacin against *L. monocytogenes* bacteremia as exemplified in the present case.

In conclusion, *L. monocytogenes* bacteremia was treated with oral levofloxacin. This treatment method might be an option for patients with *Listeria* infections; however, it is being currently used only in selected cases, such as when existing therapies are contraindicated for the patient. Although pharmacologically effective, there is little evidence of its use as a standard of care; therefore, further clinical studies are needed to establish evidence for the same.

## Ethical approval

N/A.

## Consent

Written informed consent was obtained from the patient for publication of this case report and accompanying images. A copy of the written consent is available for review by the Editor-in-Chief of this journal on request.

## Funding

This research received no specific grants from funding agencies in the public, commercial, or not-for-profit sectors.

## Authors' contributions

YI was responsible for the coordination and writing of the manuscript. All authors participated in the discussion during manuscript preparation. All the authors have agreed to publish this manuscript. All authors meet the ICMJE authorship criteria.

## CRediT authorship contribution statement

**Yo Ishihara:** Conceptualization, Writing – original draft, Writing – review & editing, Visualization, Project administration. **Kenichiro Akazawa**: Writing – review & editing.

## Conflict of Interest

The authors declare that they have no conflicts of interest.
